# The Drosophila Helicase MLE Targets Hairpin Structures in Genomic Transcripts

**DOI:** 10.1371/journal.pgen.1005761

**Published:** 2016-01-11

**Authors:** Simona Cugusi, Yujing Li, Peng Jin, John C. Lucchesi

**Affiliations:** 1 Department of Biology, Emory University, Atlanta, Georgia, United States of America; 2 Department of Human Genetics, Emory University School of Medicine, Atlanta; The University of North Carolina at Chapel Hill, UNITED STATES

## Abstract

RNA hairpins are a common type of secondary structures that play a role in every aspect of RNA biochemistry including RNA editing, mRNA stability, localization and translation of transcripts, and in the activation of the RNA interference (RNAi) and microRNA (miRNA) pathways. Participation in these functions often requires restructuring the RNA molecules by the association of single-strand (ss) RNA-binding proteins or by the action of helicases. The Drosophila MLE helicase has long been identified as a member of the MSL complex responsible for dosage compensation. The complex includes one of two long non-coding RNAs and MLE was shown to remodel the roX RNA hairpin structures in order to initiate assembly of the complex. Here we report that this function of MLE may apply to the hairpins present in the primary RNA transcripts that generate the small molecules responsible for RNA interference. Using stocks from the Transgenic RNAi Project and the Vienna Drosophila Research Center, we show that MLE specifically targets hairpin RNAs at their site of transcription. The association of MLE at these sites is independent of sequence and chromosome location. We use two functional assays to test the biological relevance of this association and determine that MLE participates in the RNAi pathway.

## Author Summary

In virtually all RNA molecules, single stranded regions undergo complementary base-pairing with neighboring regions to form double-stranded structures called stem-loops or hairpins. During the fundamental processes of transcription and translation, these RNA structures are reshaped by helicases—enzymes that separate paired regions of nucleic acids. In addition, small non-coding RNA molecules involved in regulating gene expression rely on sequences that allow them to form hairpin structures. In this paper we present evidence that the Drosophila helicase MLE, well known for its role in dosage compensation, participates in the processing of hairpin RNAs in the pathway that leads to induced RNA interference.

## Introduction

RNA hairpins are secondary structures formed by double-stranded (dsRNA) regions, known as stems, with the paired strands connected by a terminal loop. Hairpins can display a high level of heterogeneity in stem length, loop size, the number of bulges or internal loops present in the stem, and their thermodynamic properties [1]. Their function in the activation of the RNA interference (RNAi) and the microRNA (miRNA) pathways is well characterized [2]. However hairpin formation is also required in a broad spectrum of gene-expression regulatory mechanisms, including RNA editing, mRNA stability, and the specific subcellular localization of transcripts and their translation [3,4]. RNA folding can occur co-transcriptionally and transcriptional features such as pausing and elongation rate can shape this process [5–7]. A significant number of mRNAs undergo some sort of secondary structure formation *in vivo*, and cells restructure most of them through energetically driven processes [8]. Even when required to perform a specific function, the hairpins eventually need to be resolved to allow the formation of a functional RNA. In certain circumstances, the presence of hairpins in protein-coding transcripts can be harmful to the cell. For example, the tendency to form stable RNA hairpins is implicated in the pathogenesis of neurological disorders associated with trinucleotide repeats expansion [9,10]. The remodeling of RNA hairpins is achieved by association with single-stranded RNA (ssRNA) binding proteins, or by the action of helicases [11].

Helicases are ubiquitous enzymes that participate in all of the steps related to nucleic acid metabolism. Maleless (MLE), a Drosophila helicase, exhibits single-stranded RNA or DNA binding activities and is an RNA:DNA helicase/adenosine triphosphatase (ATPase) *in vitro* [12]. Orthologs of MLE, which include human RNA helicase A (RHA/DXH9), belong to the DEXH RNA helicase subfamily and are characterized by an additional domain implicated in dsRNA binding. Two specific *in vivo* functions of MLE have been reported. In the first, a mutant allele of *mle*, *mle*^*napts*^, results in a paralytic phenotype due to a splicing abnormality of the *para* mRNA in a portion of the transcript subject to RNA editing [13]. A suggested explanation of this phenotype is that the mutant MLE helicase fails to properly unwind the RNA secondary structure targeted by the ADAR enzyme (adenosine deaminase acting on RNA), compromising the access to the splicing sites retained in the hairpin. MLE’s RNA unwinding activity is also essential for its function in dosage compensation [14], where it is required for remodeling the RNA on the X (roX) RNAs’ loop structures in order to facilitate the assembly of the MSL complex [15,16]. Recently, we have obtained evidence that MLE is involved in a large number of additional regulatory steps and pathways involved in nucleic acid metabolism [17].

The ability of the MLE helicase to interact with RNA polymerase II (RNAPII) [18], and its presence at multiple actively transcribing sites on polytene chromosomes [19,20], led us to hypothesize a more general role for MLE in resolving co-transcriptionally-generated RNA hairpins. To test this hypothesis, we analyzed Drosophila lines from the Transgenic RNAi Project (TRiP) and the Vienna Drosophila Research Center (VDRC); these lines carry inducible transgenes that express hairpin RNAs specific to individual coding genes distributed across the Drosophila genome [21–24]. We determined that MLE is specifically enriched at the sites of RNAi transgene transcription, and that this association is independent of hairpin size or genomic location. Parallel functional assays establish that the MLE helicase is required for functional RNA interference *in vivo*. Our results suggest that MLE may play a broad and significant role in the structure-function relation of regulatory RNAs during development and differentiation.

In addition, our results have direct relevance to mammalian RNAi. The mammalian ortholog of MLE, RHA/DHX9, was shown to play a role in RNA interference [25,26]. This conclusion has been challenged [27]. Given that MLE and RHA are 49% identical and 86% similar, our demonstration using a genetic approach, that MLE participates in the process of RNA interference, provides support for the original conclusion that RHA plays a role in this pathway in mammals.

## Results

### MLE localizes at sites of dsRNA transcription

**Fig 1:**
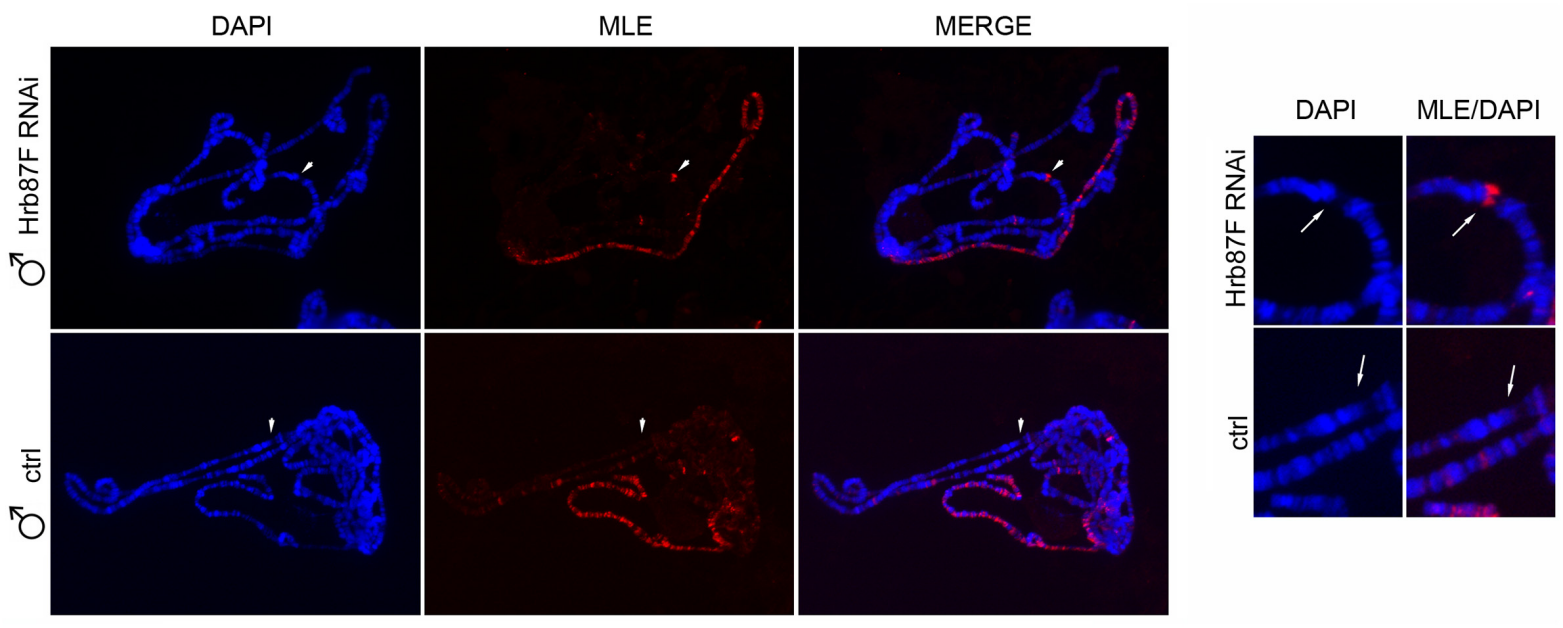
MLE is enriched at the plasmid integration site when transcription of the transgene is active. Left panel, Polytene chromosomes from male larvae expressing a dsRNA targeting *Hrb87F* (*Hrb87F* RNAi) under the induction of *Actin5C-GAL4* or larvae in which the production of the dsRNA is not activated (ctrl). MLE paints the X chromosome in both samples and is enriched at the integration site of the plasmid only in *Hrb87F* RNAi larvae. The white arrows indicate the plasmid integration site. In the right panel is a detail of the region marked by the arrows.

**Fig 2:**
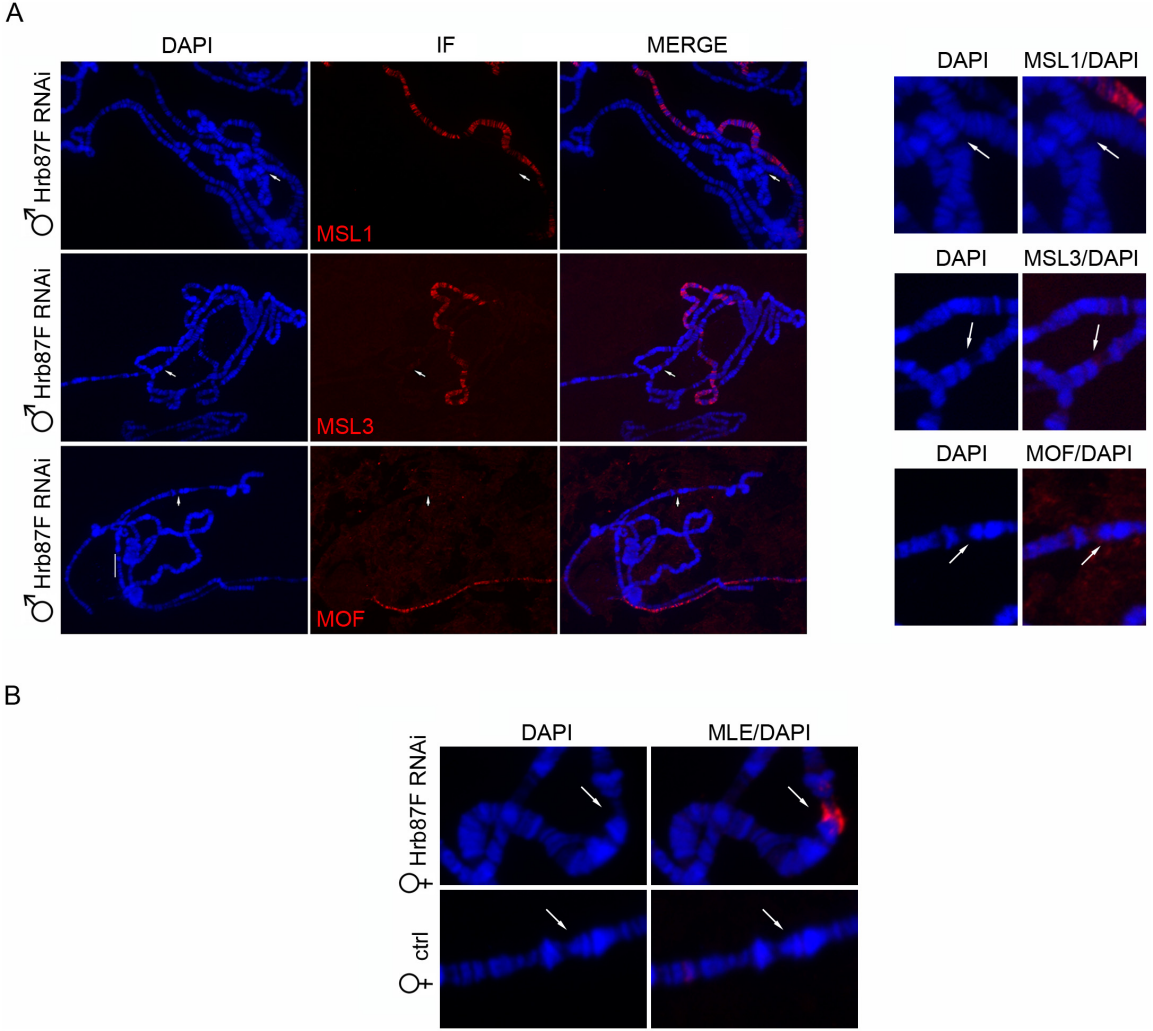
MLE localization at the integration site of the plasmid does not require the MSL complex. **(A)** Left panel, Polytene chromosomes from male larvae expressing a dsRNA targeting *Hrb87F* (*Hrb87F* RNAi) under the induction of *Actin5C-GAL4*. MSL1, MSL3 and MOF paint the X chromosome but are absent at the integration site of the plasmid indicated by the white arrows. In the right panel is a detail of the region marked by the arrows. **(B)** Polytene chromosomes from female larvae expressing a dsRNA targeting *Hrb87F* (*Hrb87F* RNAi) following induction with *Actin5C-GAL4* and larvae in which the production of the dsRNA is not induced (ctrl).

We initially analyzed MLE’s general ability to target hairpin RNAs by using RNAi stocks from the Harvard TRiP collection (flyrnai.org). These studies used an *Hrb87F* RNAi stock containing an integrated pValium1 plasmid expressing a dsRNA under the control of an inducible UAS-promoter [21]. MLE staining of male polytene chromosomes, in addition to the usual pattern on the X-chromosome and on various autosomal interbands, revealed a bright signal at the integration site of the *Hrb87F* RNAi plasmid (chromosome 3L 68A4 on the cytological map) only when its transcription was activated by an *Actin5C-Gal4* driver carried on the second chromosome (Fig 1). MLE was always present at this site (22 nuclei from 3 different larvae were analyzed). Polytene chromosome staining with MSL1, MSL3 or MOF antibodies failed to show a similar localization (at least 10 nuclei from 3 different larvae were analyzed) (Fig 2A and S1 Fig). Therefore MLE’s enrichment appears to be transcription dependent but independent of the MSL complex, this conclusion is further confirmed by the fact that MLE signal is present in all the female polytene chromosomes examined (37 nuclei from 5 larvae) at levels comparable to those observed in males (Fig 2B). Curiously, we also detected an enrichment of MLE at the telomere of the right arm of chromosome 3 in the female larvae analyzed but not in male larvae (S2 Fig). *Hrb87F* null mutants are viable but display abnormally elongated telomeres [28]; therefore, we speculate that the enrichment could be due to telomeric retrotransposon transcription, possibly correlated to the developmental stage of the larvae. To our knowledge, this type of targeting has never been reported in the literature and we never observed it in any of our other experiments. This aspect, although interesting, lies outside the focus of the present work. To rule out the formal possibility that the chromosome 2 Gal4 activator used was responsible for the observed effects on MLE localization, we crossed the *UAS*-*Hrb87F* RNAi flies with flies carrying an *Actin5C-Gal4* transgene on the third chromosome. The progeny of this cross also showed a clear localization of MLE at the plasmid integration site (S3 Fig). Surprisingly, no signal on the 3R telomere was detected in these larvae.

### MLE enrichment at sites of dsRNA transcription is sequence and chromosome location independent

**Fig 3:**
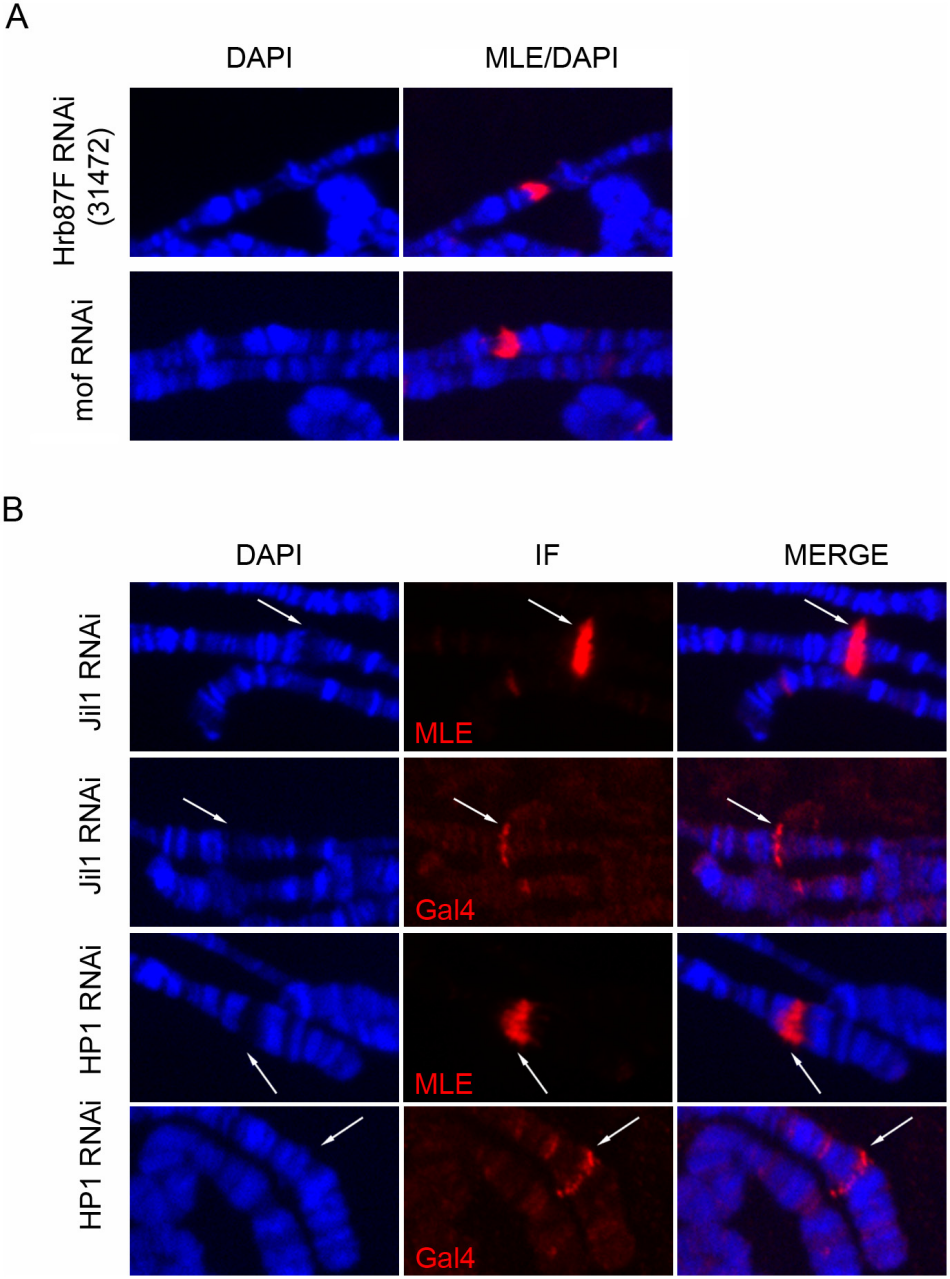
MLE targets sites of dsRNA transcription in a sequence and chromosome location independent manner. **(A)** MLE staining of polytene chromosomes from female larvae expressing either a *mof* dsRNA or an *Hrb87F* dsRNA construct from a pValium1 insertion of TRiP line collection following induction with *Actin5C-GAL4*. **(B)** GAL4 and MLE staining of polytene chromosomes from female larvae expressing dsRNA constructs integrated respectively at Chr2L 30B3 (*Jil1* RNAi) and Chr2L 22A5 (*Hp1* RNAi) and induced by *Actin5C-GAL4*.

In order to determine whether the MLE localization is sequence specific, we tested MLE localization in the background of two additional TRiP pValium1 lines, one expressing a dsRNA targeting a different portion of the *Hrb87F* gene and one targeting *mof*, the gene that encodes the histone acetyl transferase present in the MSL complex in males, but that is also found in both sexes [29]. To avoid any ambiguity due to MLE’s function in dosage compensation, we restricted these analyses to female larvae. As can be seen in Fig 3A, MLE is highly enriched at the plasmid integration site in both the second *Hrb87F* RNAi line and the *mof* RNAi line, indicating that its localization is independent of the sequence of the dsRNA being transcribed. Moreover, the successful MOF knock down (S4 Fig) excludes a role of this protein in MLE localization. We also tested if any sequence included in the inserted transgene, other than the dsRNA, was recruiting MLE. In the pValium1 lines, the transcriptional unit under the control of the UAS-promoter contains an intron of the *white* gene between the two inverted repeats. To investigate whether MLE specifically targeted the RNA sequence of this intron, we took advantage of the TRiP lines transformed with pValium10 [22]. This plasmid is integrated in the same genomic site as pValium1 but contains several unique features including a *fushi tarazu* gene intron replacing the *white* gene intron. We tested three different lines and all three of them recruited MLE (S5 Fig), arguing that MLE is not recruited by transcribed sequences in the *white* intron.

pValium plasmids contain the *vermillion* gene as a selectable marker and the attP landing site is flanked by a *yellow* gene. Because the transcriptional status of these genes does not vary with the presence of a GAL4 activator we considered rather unlikely that they may be responsible for the MLE enrichment. Nevertheless, to test this possibility, we used two lines from the VDRC RNAi collection UAS-Jil1 dsRNA and UAS-Hp1 dsRNA, in which the transgenes have been inserted into different sites in the genome using different targeting techniques. The Hp1 dsRNA construct is randomly integrated in the genome *via* a P-element-mediated germ line transformation; it does not contain either the *vermillion* or the *yellow* gene and uses the *white* gene as a selectable marker. The Jil1 dsRNA construct is inserted in chromosome 2L band 30B3 *via* site-specific recombination; it does not contain the *vermillion* gene, the *white* gene is used as a marker and the landing site is flanked by a *yellow* gene. In order to map the genomic integration sites for the Hp1 and Jil1 RNAi transgenes and correlate them with the association of MLE, we stained polytene chromosomes bearing these transgenes in combination with the Actin5C-GAL4 driver with a GAL4 antiserum. We used the DAPI pattern to precisely map the MLE and GAL4 signals and determined that MLE is enriched at the integration site of the plasmids when transcription is activated (Fig 3B).

### MLE specifically targets hairpin RNAs at their site of transcription

**Fig 4:**
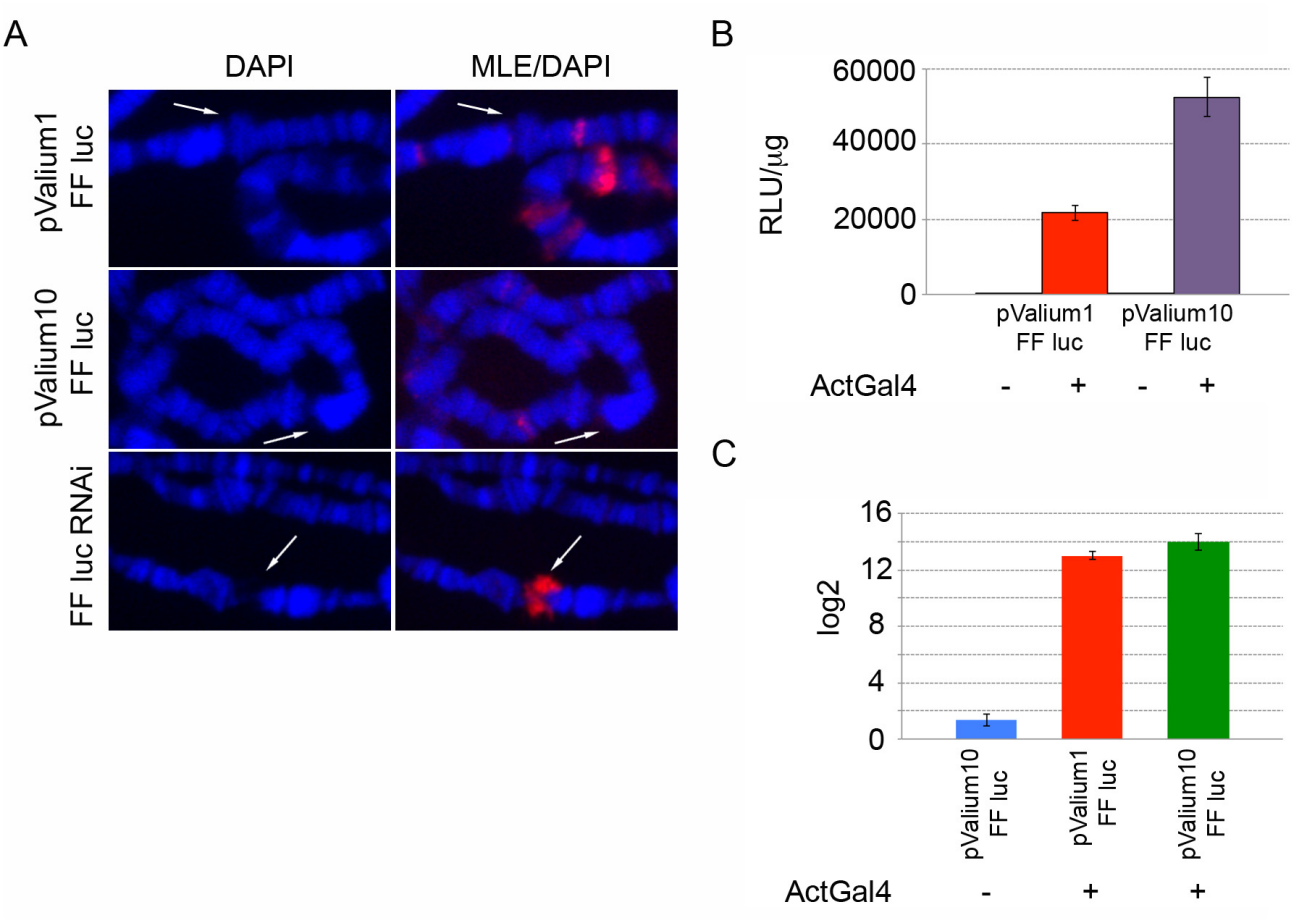
MLE is not recruited by high levels of expression. **(A)** MLE staining of polytene chromosomes from female larvae expressing either the luciferase gene or a dsRNA targeting the luciferase gene following induction with *Actin5C-Gal4*. MLE is present at the integration site of the plasmid when the dsRNA is transcribed but not when the luciferase gene is expressed. White arrows indicate the integration site of the plasmid. **(B)** Luciferase assay in larvae carrying ActGal4-induced and non-induced pValium1 and pValium10-mediated luciferase gene inserts. High levels of luciferase are observed after induction. **(C)** qRT-PCR analysis of luciferase gene transcript levels in larvae carrying ActGal4-induced and non induced pValium1 and pValium10-mediated luciferase gene inserts. Luciferase gene expression was normalized using *pka* gene and the results are expressed in terms of fold difference relative to the basal transcript levels observed in pValium1-luciferase sample without induction. The results are the average of three independent biological replicates.

MLE is recruited to a pool of highly expressed developmentally regulated genes [20]; therefore, the observed MLE enrichment could be due to the high level of transcription caused by GAL4 activation, rather than by the features of the transcript. To test this possibility we used two control stocks from the TRiP collection that express the luciferase gene in the presence of GAL4; the luciferase genes, inserted either in a pValium1 or a pValium10 plasmid, lack a hairpin-generating sequence. In this case, although MLE is seen at a few sites on the polytene chromosomes, we did not observe any binding at the integration site of the plasmid (Fig 4A), confirming that this enzyme is specifically recruited at sites of hairpin RNA synthesis. The level of luciferase gene expression was verified by luciferase assay and qRT-PCR (Fig 4B and 4C). We also tested a stock expressing a dsRNA against the luciferase gene and observed the presence of a strong MLE signal at the site of hairpin transcription. This result excludes the possibility that the absence of MLE binding to the overexpressed luciferase gene is due to its inability to recognize that specific gene sequence; rather it is due to the absence of a hairpin in the transcript (Fig 4A).

**Fig 5:**
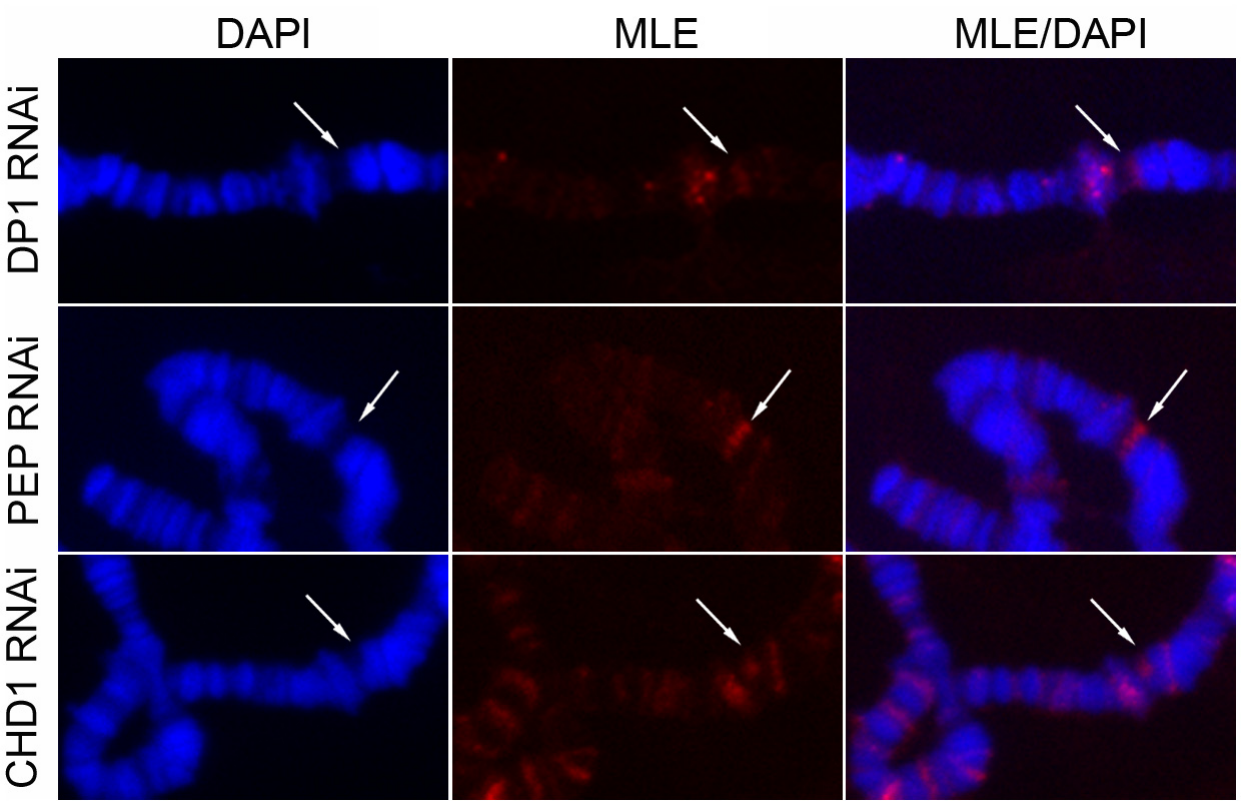
MLE targets shRNA at their site of transcription. MLE staining of polytene chromosomes from female larvae expressing short hairpin RNAs after induction with *Actin5C-GAL4*.

All the TRiP and VDRC RNAi lines tested so far expressed long hairpin RNAs (lhRNAs) with stems ranging from 331 to 570 bp (S1 Table). We next asked if the size of the dsRNA was a critical feature for MLE recruitment. We analyzed three different TRiP lines that express short hairpin RNAs (shRNAs) with stems of 21 nucleotides [23]. We found that MLE was present in all of them at the site of the RNAi transgene although the enrichment was not as robust as in the lines expressing lhRNAs (Fig 5). This difference may be due to a weaker affinity of MLE for short hairpin RNAs or to a faster release of the helicase from a short target.

**Fig 6:**
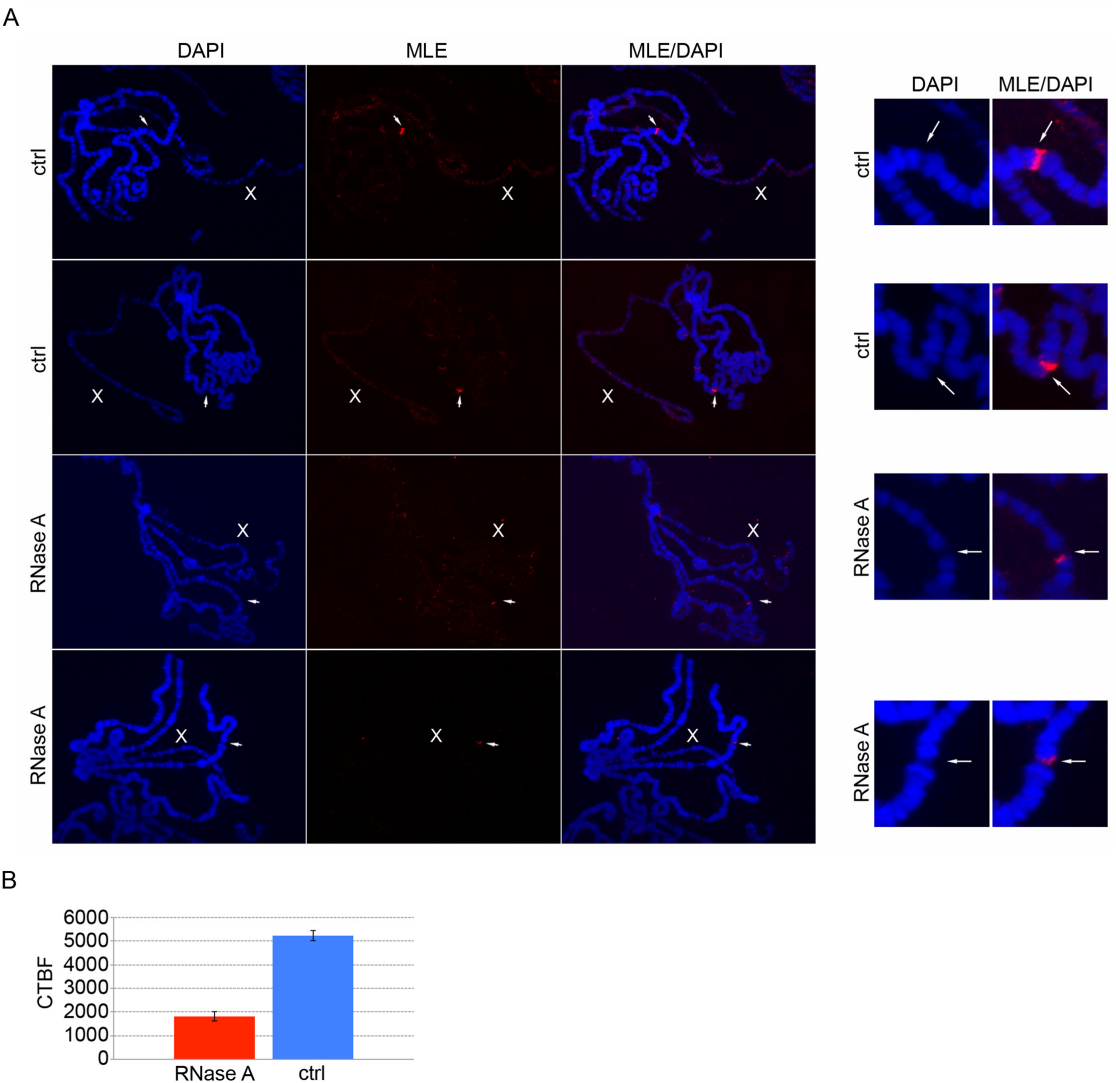
RNase treatment strongly reduces MLE signal at the integration site of the plasmid. **(A)** Left panel, MLE staining of polytene chromosomes from male larvae expressing a dsRNA targeting *Hrb87F* after induction with *Actin5C-GAL4*. The incubation of the salivary glands in RNase A (RNase A) perturbs MLE localization at the integration site of the plasmid while in the absence of RNase A (ctrl) the MLE signal is still highly enriched. The white arrows indicate the plasmid integration site. In the right panel is a detail of the region marked by the arrows. **(B)** Quantitative analysis of fluorescence levels. MLE signal at the integration site of the plasmid, expressed in terms of corrected total band fluorescence (CTBF), is significantly reduced after RNase A treatment (p value <0.001). The analysis was performed on 33 polytene chromosomes treated with RNase A and 11 control chromosomes.

It is possible that MLE is recruited to the integration site of the plasmid by a DNA alternative structure formed during the inverted repeat’s transcription and not by the architecture of the transcript. Therefore, in order to establish the RNA-specific nature of the binding, we incubated polytene chromosomes with RNase A. We chose to perform this experiment on male larvae in order to use the RNA dependent localization of MLE on the X chromosome as a positive control for the RNase treatment. As previously reported [30], cell permeabilization steps necessary to introduce RNAse into unfixed cells partially destabilize MLE binding to the X chromosome, but they do not compromise MLE binding to the integration site of the plasmid (Fig 6). Following RNase treatment, MLE is strongly reduced at the hairpin RNA transcription site and is completely released from the rest of the X chromatin. Co-staining of MLE and MSL1, a protein not affected by RNase treatment, confirms the specific effect of RNase on MLE binding (S6 Fig). This result indicates that MLE binding at the site of hairpin RNA synthesis, while requiring the presence of RNA, is structurally different from its binding to the X chromosome.

A parallel treatment with RNase III, which specifically cleaves dsRNA, did not result in a measurable effect on MLE localization (S7 Fig). However due to the lack of a positive control we are not able to discriminate between a resistance to the treatment and a technical issue.

**Fig 7:**
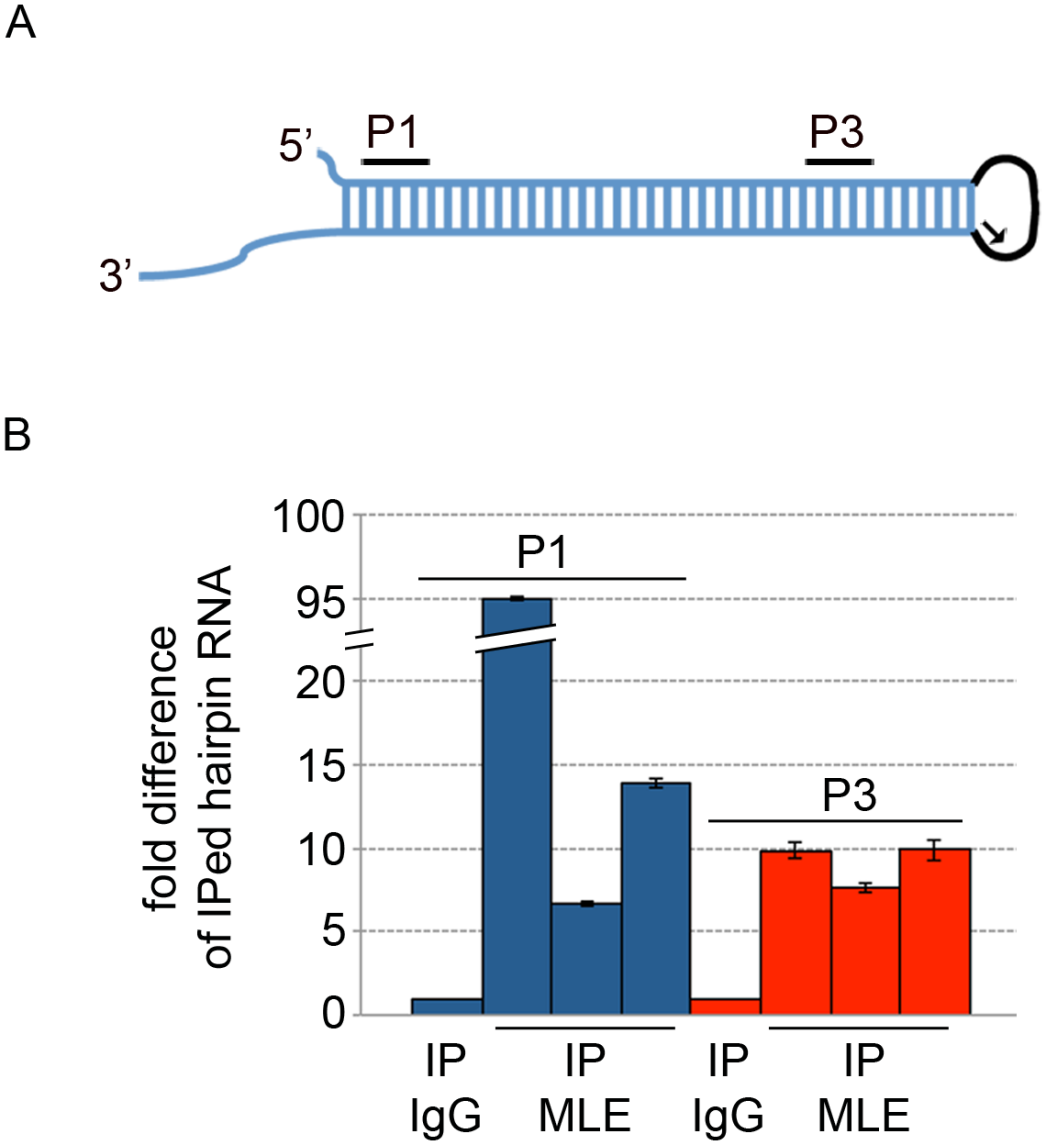
MLE physically interacts with RNA hairpins. **(A)** Schematic representation of the hairpin RNA generated by pValium1-*Hrb87F* RNAi induction. In blue is the stem formed by the inverted repeats, in black is the *white* intron. The arrow indicates the primer used to obtain the cDNA and the two fragments analyzed by qPCR (P1 and P3) are indicated by the black lines **(B)** RIP experiment from extracts of female larvae expressing a dsRNA targeting *Hrb87F* after induction with *Actin5C-GAL4*. The results are expressed in terms of fold difference between MLE-associated RNA and IgG-associated RNA normalized for the starting material. Three independent biological replicates are presented for P1 and P3 primer pairs.

To provide further evidence that MLE physically binds to RNA hairpins, we performed an RNA immunoprecipitation (RIP) experiment followed by a qRT-PCR reaction. Extracts from larvae expressing Hrb87F lhRNA were used for immunoprecipitation with either anti-MLE or generic IgG antibodies. The immunoprecipitated RNA was isolated and quantified by gene-specific qRT-PCR with IgG as a negative control. In this reaction, the cDNA was obtained using a primer targeting the loop structure formed by the *white* intron and it was then amplified with two different primers sets targeting the stem region (Fig 7). Hrb87F hairpin was found reproducibly enriched in MLE-associated RNAs over IgG-associated RNAs. While this experiment demonstrates that MLE binds the RNA in question, specificity was not assessed.

### MLE participates in the RNAi pathway

**Fig 8:**
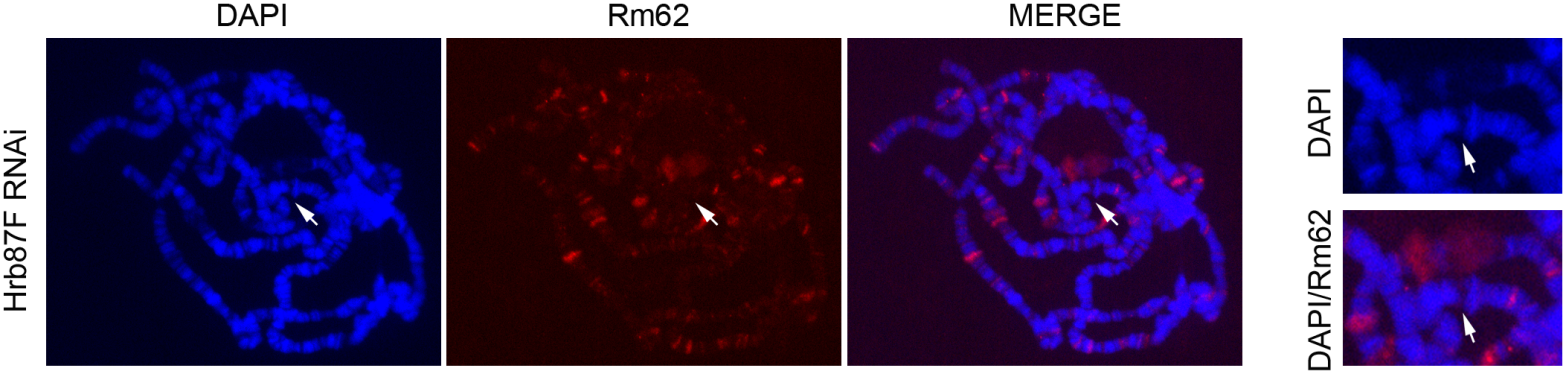
Rm62 RNA helicase is not enriched at sites of hairpin RNA transcription. Left panel, Rm62 staining of polytene chromosomes from female larvae expressing a dsRNA targeting *Hrb87F* after induction with *Actin5C-GAL4*. The white arrows indicate the plasmid integration site. The right panel shows a detail of the region marked by the arrows.

It is possible that a highly expressed hairpin RNA attracts a broad range of RNA binding proteins including RNA helicases in a non-specific manner. To address this possibility, we tested the recruitment of the Rm62 RNA helicase to the integration site of the plasmid. Like MLE, Rm62 is a member of the DExD/H subfamily of helicases; it localizes at actively transcribed genes, such as developmentally regulated puffs, and is implicated in multiple biological processes among which chromatin insulation, RNA export, splicing and transcriptional repression [31–33]. More importantly, it takes part in the RNAi pathway [34,35]. Furthermore the human homologue of Rm62, DDX17, binds pri-miRNAs stem-loop structures [36]. Polytenes staining from larvae expressing *Hrb87F* hairpin RNA did not show any binding of Rm62 helicase at the integration site of the plasmid, although the protein is present at other sites such as developmental puffs (Fig 8). A co-staining experiment revealed that MLE and Rm62 co-localize on certain puffs and sites; however this was never the case for the induced hairpin transcription site (S8 Fig). A weak background signal of Rm62 at the integration site of the plasmid, present in the co-stained polytene chromosomes but not observed in the chromosomes stained only with Rm62, is probably due to bleed-through of the strong MLE signal or to an antibody cross-reaction. The above results indicate that the hairpin transcription is specifically recruiting MLE rather than generically attracting RNA helicases.

**Fig 9:**
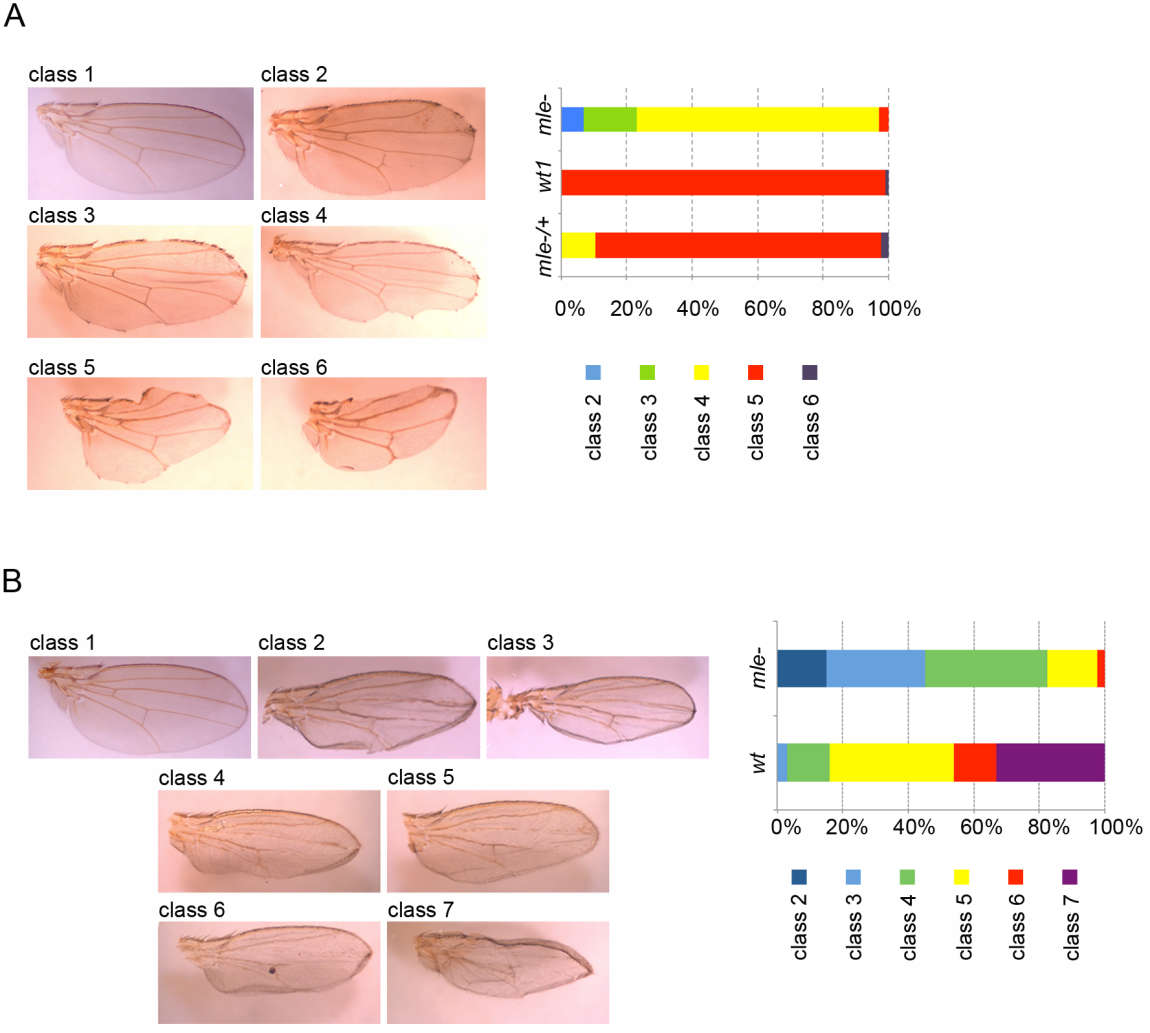
MLE mutation affects RNAi efficiency. **(A)** Wing phenotypes from female flies in which Notch dsRNA expression is induced by *C96-GAL4*. Class1: wild type; class 2: missing margin bristles and absent notching; class 3: moderate notching; class 4: extensive notching; class 5: missing most of the wing margins; class 6: complete lack of margins and reduced wing blade. The chart on the right side of the figure represents the *Notch* RNAi wing phenotypes distribution in *wild type*, *mle* homozygous mutant and *mle* heterozygous mutant background. The results are the sum of three to six independent crosses per genotype. **(B)** Wing phenotypes from female flies in which Egfr dsRNA expression is induced by *ms1096-GAL4*. Class1: wild type; class 2: all the veins present; class 3: absent anterior cross vein (acv) or presence of a partial longitudinal vein; class 4: absent acv plus one partial longitudinal vein; class 5: absent acv plus two partial longitudinal veins; class 6: acv vein absent plus one longitudinal vein absent plus one partial longitudinal vein; class 7: most of the veins absent. The chart on the right side of the figure represents the *Egfr* RNAi wing phenotypes distribution respectively in *wild type* and *mle* homozygous mutant background. The results are the sum of four to five independent crosses per genotype.

The ability of MLE to target hairpin RNAs at their sites of transcription and our published observations that MLE interacts with the components of the RNAi machinery, Argonaute 2 and Dicer-2 [17], led us to investigate the possibility that MLE is a required element of the RNAi pathway. To this end, we compared the efficiency of a *Notch* RNAi knockdown in a *wild type* vs. *mle* mutant background using the recessive null allele *mle*^*1*^. Notch dsRNA expression in the wing margins, induced by a *C96-Gal4* driver, leads to a broad range of adult phenotypes, giving rise to 6 classes, from the absence of a few margin bristles and mild notching, to a complete lack of margins and profound reduction in size of the wing blade [22,23]. In a wild type background female population raised at 25°C, C96-driven expression of the *Notch* RNAi resulted in a significant reduction of the wing margin (class 5) in nearly 100% of the 260 wings examined, with the remaining wings showing an even more severe phenotype (class 6) (Fig 9A). This result is concordant with those reported by Ni et al., 2011 [23]. Heterozygosity for the *mle*^*1*^ allele had little effect on Notch-RNAi induced wing phenotypes; the 125 wings scored exhibit a phenotypic distribution that closely resembles the distribution previously observed in the wild type background (Fig 9A), suggesting that RNAi efficiency is not profoundly impaired by halving the genetic dose of MLE. Approximately 10% of the wings retained most of the wing margin (class 4), perhaps due to a mild *mle*^*1*^*/+* heterozygote effect or to some unknown influence of the genetic background. Albeit small, this difference is nevertheless significant (Chi-square value = 29, p value <0.001). Critically, in an *mle*^*1*^*/mle*^*1*^ mutant background we observed a substantially less severe *Notch* RNAi phenotype: all 115 wings analyzed retain most of their margins, with 70% of them exhibiting extensive notching (class 4) and approximately 30% showing mild or no notching (class 2 and 3). In this case, as well, the difference is highly significant (Chi-square value = 358, p value <0.001). To test whether the effect of MLE on the wing phenotype induced by *Notch* RNAi was due to the specific *mle* allele used, females with an *mle*^*1*^*/mle*^*Y203*^ heteroallelic combination were obtained. Here again the absence of MLE leads to an improvement in wing development (S9 Fig) that is similar to the improvement obtained with the homozygous *mle*^*1*^ allele (Chi-square value = 0.68, p value = 0.71). These results suggest that MLE normally enhances the efficiency of the RNA machinery.

In order to demonstrate that this effect is reproducible with other dsRNAs, we performed a similar experiment by driving *Egfr* (epidermal growth factor receptor) dsRNA synthesis in the wing with the *ms1096-GAL4* driver. *Egfr* is involved in wing vein development [37], and its knockdown in the developing wing with *ms1096-GAL4* leads to profound adult vein defects. By classifying the observed vein phenotypes according to the number of veins affected we defined the 7 classes listed in Fig 9B. In a wild type background more than 80% of the *ms1096-Egfr* RNAi wings examined (n = 92) were concentrated in the 3 classes with the most severe phenotypes (classes 5, 6 and 7) and no wings in class 2 (the least severe phenotype) were detected. In contrast, in an *mle*^*1*^*/mle*^*1*^ mutant background, more than 80% of the *ms1096-Egfr* RNAi wings (n = 86) fall in the 3 mildest phenotypic classes (classes 2, 3 and 4); no wings belonging to class 7 were found. The observed effect of the absence of MLE on the *Egfr* RNAi phenotype is significant (Chi-square value = 88, p value <0.001).

## Discussion

MLE is a Drosophila helicase required for dosage compensation in males. *In vitro*, it was shown to unwind short double-stranded RNA or RNA/DNA substrates [12]. An early indication of its *in vivo* RNA remodeling properties was offered by the phenotype of the *mle*^*napts*^ allele, which suggested that MLE may play a role in resolving loop structures in the *para* gene transcript [13]. Such a function was validated by the demonstration that MLE remodels the roX RNA hairpins during the assembly of the MSL dosage compensation complex [15,16]. As MLE has been recently implicated in a variety of regulatory steps and pathways [17], we asked whether it might have a broader role and participate in other pathways that involve the biogenesis or function of hairpin RNAs. To this end, we have carried out a series of cytological, molecular and genetic investigations, and have obtained evidence that MLE targets RNA hairpin structures at their site of transcription. This activity is independent of the sequences forming the hairpins or of the genomic location where their synthesis occurs. The MSL complex does not take part in this process.

Of some interest is a set of published observations that may suggest a link between an effect of MLE in females and some observed phenotypes of mutants in the RNAi pathway. Adult females homozygous for an *ovo* gene null allele have no or very few germ cells. Introduction of the *mle*^*1*^ mutation largely restores the germ line in these females [38]. Mutations in the genes that encode different components of the miRNA biogenesis pathway–*Argonaute 1* (*Ago 1*), *Dicer-1 (*Dcr-1), *pasha* and *Drosha*—interrupt germ-line cell division and oocyte formation [39]. Connecting these two experimental dots is our observation that MLE co-immunoprecipitates with Argonaute 2 (Ago 2) and Dicer-2 [17]. In males, MLE is the only member of the MSL dosage compensation complex present in germ cells where it is not specifically associated with the X chromosome [40]. Recently, the RNAi pathway was shown to impact spermatogenesis and male fertility through the generation of endo-siRNAs from endogenous hpRNAs [41]. Although MLE has not been directly implicated in male gamete formation, it may be suggestive to note that the induced lhRNAs used in our experiments are more similar to endo-siRNAs precursors than to miRNAs precursors.

MLE’s targeting the transcripts of RNAi transgenes indicates that it may play an active role in the RNA interference pathway. In support of such a potential role, we have demonstrated that MLE is required for optimal RNAi efficiency. RNA helicase A (RHA/DHX9), the mammalian ortholog of MLE, plays a role in RNA interference. RHA associates with Ago 2 and Dicer in human cells [25], it is a component of the RISC complex, and it facilitates the assembly of the complex [26,42]. It is possible that MLE acts in a similar fashion, however our results indicate that MLE binds hairpin structures in primary transcripts and, therefore, appears to play a function at an earlier step in the biogenesis of interfering RNAs. A possible explanation is that the transcripts produced by RNAi transgenes may not require the action of the Drosha RNase because their sequence forms hairpins that are already structurally similar to conventional pri-miRNAs. Such a situation exists in the case of mirtrons, miRNAs that are produced from introns by the splicing machinery and that bypass Drosha cleavage [43]. In fact, the processing of endogenous long hairpin RNAs does not involve Drosha [44]. MLE may bind to the RNAi hairpins in order to facilitate their transfer to the cytoplasm, and it may, in a manner similar to RHA, also participate in the formation of the RISC complex. An alternative explanation is that MLE might resolve improper folding of the newly transcribed hairpin RNAs allowing them to enter the RNAi pathway.

Another possibility is that MLE interferes with the editing activity of the ADAR enzyme. ADAR and the RNAi pathway are in an antagonistic relationship, likely due to the fact that they compete for the same substrates [45,46]. ADAR deamination of adenosines to inosine at multiple sites can lead to reduced complementarity and dsRNA instability limiting the synthesis of productive siRNA. MLE could physically block ADAR’s binding to the hairpins or it could unwind the structure to compromise ADAR’s recruitment.

All the discussed scenarios are not mutually exclusive. Therefore, it is not unreasonable to consider that MLE could play a role in the Drosophila immune response against inverted repeat viruses, as well as in retrotransposon silencing and in the regulatory pathways controlled by the recently identified endogenous hairpin RNAs [41,44]. Moreover, the finding that not only long hairpins but also short hairpin RNAs recruit MLE suggest that MLE might be involved in the microRNA pathway as well.

## Material and Methods

### Fly stocks and genetic crosses

TRiP collection stocks 31244 and 31472 (*Hrb87F* RNAi), 31401 (*mof* RNAi), 32872 (*Dp1* RNAi), 32944 (*Pep* RNAi), 34665 (*Chd1* RNAi), 28981 (*Notch* RNAi), 25781 (*Egfr* RNAi), 31603 (*Luciferase* RNAi), 35789, and 35788 (Luciferase overexpression) were obtained from the Bloomington Drosophila Stock Center (BDSC). TRiP RNAi stocks 26772 (*CG14962* RNAi), 25993 (*CG4617* RNAi) and 31922 (*CG3838* RNAi) were a gift from B. Yedvobnick and are available at BDSC. Stocks v107001 (*Jil1* RNAi) and 31994 (*Hp1* RNAi) were obtained from the Vienna Drosophila Resource Center (VDRC). The *C96-GAL4*, (stock 43343) and the *Actin5C-Gal4* (stock 3954) drivers were obtained from the BDSC and the *ms1096-GAL4*,*UAS-GFP* driver was a gift from K. H. Moberg.

Flies homozygous for the RNAi constructs were crossed with flies containing *Actin5C-Gal4* driver balanced either with *CyO-GFP or TM6B*, third instar larvae lacking the GFP or Tubby markers were selected for polytene staining while larvae showing the markers were used as controls.

To test the effect of MLE on the wing phenotype induced by *Notch* RNAi, *mle*^1^/*CyO*; *UAS*-*Notch dsRNA*/*TM6B* females were crossed to *mle*^1^/*CyOGFP*; *C96-Gal4/TM6B* males to obtain *mle*^1^/*mle*^1^; *UAS-Notch dsRNA/C96-Gal4* females; *mle*^1^/*CyO*; *UAS-Notch dsRNA/TM6B* females were crossed to *+/CyOGFP; C96-Gal4/TM6B* males to obtain *mle*^1^*/+; UAS-Notch dsRNA/C96-Gal4* females; control females were *+/+; UAS-Notch dsRNA/C96-Gal4*. To test whether the effect was due to the specific allele used, females with the *mle*^1^/*mle*^Y203^ heteroallelic combinations were obtained. To test the effect of MLE on the wing phenotype induced by *Egfr* RNAi, *+/+; mle*^*1*^*/CyO UAS-Egfr dsRNA/TM6B* females were crossed to *ms1096-GAL4*,*UAS-GFP/Y; mle*^*1*^*/CyO; +/+* males to obtain *ms1096-GAL4*,*UAS-GFP/+; mle*^*1*^*/mle*^*1*^; *UAS-Egfr dsRNA/+* females; control females were *ms1096-Gal4*,*UAS-GFP/+; +/+; UAS-EGFR dsRNA/+* females. The description of the *CyO* and *TM6B* balancer chromosomes and of the other genetic elements in these crosses can be found in FlyBase (flybase.org). Wings from adult females were removed and mounted in Euparal mounting medium (BioQuip Products). The Chi-square test was used for statistical analysis.

### RNA extraction and qRT-PCR

RNA was isolated from 10 larvae per sample using the Qiagen RNeasy mini-kit following manufacturer’s instructions. iScript One-step RT-PCR kit with SYBR Green (BIO-RAD) was used for the real-time, reverse transcription-PCR. Transcription measurements were normalized to the Pka-C1 transcript and the ddCt method was used to calculate the fold difference. The results of three independent biological replicates were averaged. The primers used to detect Luciferase are: forward 5’-AGGTTCCATCTGCCAGGTATCAG-3’ and reverse 5’-ACACACAGTTCGCCTCTTTGATTAAC-3’. The primers used to detect Pka-C1 are: forward 5'-TTCTCGGAGCCGCACTCGCGCTTCTAC-3' and reverse 5'-CAATCAGCAGATTCTCCGGCT-3'

### Luciferase assay

Larvae were frozen at -80°C, thawed and homogenized in 100 μl of Promega Passive lysis buffer. The lysates were then frozen at -80°C and thawed at 37°C for three times then were spun at 13000 rpm for 5’. 20 μl were used for the luciferase assay according to manufacturers protocol. Relative light units (RLU) were normalized for protein content. The results are the average of three independent biological replicates

### RIP

50 of larvae per sample were homogenized in 1ml of lysis buffer containing 25 mM Tris-HCL pH 7.5, 300 mM NaCl, 2mM MgCl2, 5mM DTT, 0.5% NP40, protease inhibitor (Roche, Cat#05892791001), and RNasin (Promega, Cat# N2515) and incubated on ice for 10 min. Lysates were then sonicated at 0-3W and 2V for 27 seconds x 4 on ice before spinning at 14000 rpm for 10’ at 4°C. The supernatant was pre-cleared with Dynabeads Proteinase G (Life Technologies, Cat# 10009D) for 1h at 4°C. 10% of the pre-cleared lysates was saved for protein quantitation for final normalization and the remaining 90% then incubated with 50 μl of Dynabeads Protein G slurry and equal volumes of either pAb-anti-MLE, or generic anti-mouse IgG (Jackson IR) antibody as IP control, for 1-2h at 4°C. After being washed five times with lysis buffer, the IPed beads were digested with RNase-free DNase RQ1 (Promega, Cat#6106) and RNasin in digestion buffer at 37°C for total 30min to remove genomic DNA resulting from non-specific binding. After washing with lysis buffer, the DNase-digested beads were treated with Trizol (Life Technologies, Cat# 15596018) and chloroform to extract the IPed RNA that was precipitated with glycogen, 3M NaAc, and isopropanol O/N at -80°C followed by spinning down at maximum speed, at 4°C, for 20 min. After washing with 75% ethanol, the IPed RNA pellets were resuspended in nuclease-free water for reverse transcription using SuperScript III reverse transcriptase (Cat# 18080–44, Life Technologies) to generate the first strand cDNAs using the primer 5’-TGAGTTTCAAATTGGTAATTGGACCCT -3’. The first strand cDNAs were applied as templates for real-time PCR using SYBR green and the following set of primers were used: Hrb87F-P1 forward 5’-TGCTTGGCAATAGCCTTCTTCA-3’, Hrb87F-P1 reverse 5’-TCGATGACTACGATCCCGTTGACA-3’, Hrb87F-P3 forward 5’-GCATTGTCGATCATGTACGACT-3’, Hrb87F-P3 reverse 5’-CTTCGGTTTCATCACGTACT-3’. Three independent biological replicates are presented for P1 and P3 primer pairs.

The MLE-IP efficiency and the lhRNA enrichment in the MLE-IPed RNAs were calculated based on two levels of normalizations. First, concentrations of the crude protein in the starting lysates were normalized to make sure equal amount of protein were used for both the MLE-IP and the IgG-IP. Second, the average Ct numbers from the IgG-IPs were used for normalization to compare the lhRNA enrichment by the MLE-IP relative to the IgG-IP, expressed in terms of fold difference 2^-dCt.

### Polytenes chromosome squashes and immunostaining

Salivary glands from third instar larvae were dissected in PBS buffer and fixed for 10’ with 2% formaldehyde in 45% acetic acid. After squashing, the slides were frozen in liquid nitrogen, washed twice in TBS-Triton 0.1% and incubated in TBS-Triton containing 5% milk for 30’. Polytenes preparations were then incubated overnight with primary antibody in humid chambers at 4°C, washed twice in TBS-Triton 0.1% and incubated with a secondary antibody at room temperature for 1hr. After washing twice in TBS-Triton 0.1% the slides were mounted using VECTASHIELD mounting medium with DAPI. For the RNase A and RNase III treatments, salivary glands were dissected in PBS, transferred to PBS-Triton 0.1% for 5’ and incubated in PBS with or without 0.5 mg/ml RNase A (Qiagen) for 10’. Primary antibodies were used at the following concentrations: MSL1 (1:300), MSL3 (1:50), MLE (1:300), MOF (1:50), GAL-4 by Babco/BioLegend (1:50), Rm62, a gift from E. Lei, (1: 100). For the co-staining experiments an MLE antibody raised in Guinea pig was used at 1:50. The secondary antibodies are: Rodamin Red-X anti-Rabbit (Jackson IR) (1:500) and FITC anti-Guinea pig (Jackson IR) (1:500).

The quantitative fluorescence analysis of polytenes treated with RNase A was performed using imageJ software. For each polytene chromosome analyzed, three areas adjacent to the fluorescent band were selected to measure background levels, the mean background fluorescence was multiplied by the area of the fluorescent band and subtracted from the integrated density of the band in order to obtain the corrected total band fluorescence (CTBF). The T-Test was applied for statistical validation.

## Supporting Information

S1 Fig The MSL complex does not localize at the integration site of the plasmid. Polytene chromosomes from male larvae expressing a dsRNA targeting *Hrb87F* (Hrb87F RNAi). Co-staining with Guinea-pig anti-MLE and respectively rabbit anti-MSL1, MSL3 or MOF. The white arrows indicate the integration site of the plasmid.
(PDF)

S2 Fig MLE localizes at 3R telomere when Hrb87F is knocked down. Polytene chromosomes from female larvae expressing a dsRNA targeting *Hrb87F* (Hrb87F RNAi). MLE staining results in a clear signal at the integration site of the plasmid, indicated by a white arrow, and at the 3R telomere, indicated by an asterisk.
(PDF)

S3 Fig MLE localization at the integration site of the plasmid is independent of the driver used. Polytene chromosomes from female and male larvae in which the expression of a dsRNA targeting *Hrb87F* (Hrb87F RNAi) has been induced by an Act5C-GAL4 driver on the third chromosome. MLE staining results in a clear signal at the integration site of the plasmid expressing the dsRNA. The white arrows indicate the plasmid integration site.
(PDF)

S4 Fig MOF knock down. Polytene chromosomes from male RNAi larvae co-stained with anti-MLE and anti-MOF. MLE is present at the integration site of the plasmid (white arrow) and at the X-chromosome in both Hrb87F RNAi and MOF RNAi larvae. MOF is present on the X-chromosome of Hrb87F RNAi larvae while it is completely absent in MOF RNAi larvae, indicating that it was successfully knocked down.
(PDF)

S5 Fig MLE is not recruited by the *white* intron. MLE staining of polytene chromosomes from female larvae expressing three different dsRNA inserted in a pValium10 plasmid.
(PDF)

S6 Fig RNaseA treatment affects MLE localization at the integration site of the plasmid. **(A)** MLE and MSL1 co-staining of polytenes chromosome from male larvae expressing a dsRNA targeting *Hrb87F* induced by an Act5C-GAL4 driver. Incubation with RNaseA reduces MLE’s enrichment at the site of hairpin transcription. The white arrows indicate the integration site of the plasmid. **(B)** Magnification of the area indicated by the arrow in portion (A) of the figure. **(C)** Quantitative analysis of fluorescence levels. MLE signal at the integration site of the plasmid, expressed in terms of corrected total band fluorescence (CTBF), is significantly reduced after RNase A treatment (p value <0.001). The analysis was performed on 6 polytene chromosomes treated with RNase A and 8 control chromosomes.
(PDF)

S7 Fig RNase III treatment does not perturb MLE localization at the integration site of the plasmid. MLE staining of polytenes chromosome from male larvae expressing a dsRNA targeting *Hrb87F* induced by an Act5C-GAL4 driver. Incubation with RNase III does not appear to affect MLE’s enrichment at site of hairpin transcription. The white arrows indicate the integration site of the plasmid.
(PDF)

S8 Fig MLE and Rm62 co-localize at developmental puffs but not at the hairpin transcription site. MLE and Rm62 co-staining of polytene chromosomes from female larvae expressing a dsRNA targeting *Hrb87F* induced by an Act5C-GAL4 driver. **(A)** MLE is highly enriched at the integration site of the plasmid (indicated by the white arrow) while Rm62 does not appear to be enriched at the same site. **(B)** MLE and Rm62 are both enriched at developmental puffs.
(PDF)

S9 Fig *Notch* RNAi efficiency in *mle* mutant heteroallelic combination. Wing phenotypes from female flies, raised at room temperature, in which Notch dsRNA expression is induced by *C96-GAL4*. No difference is observed between the homozygous mutant *mle*^*1*^*/mle*^*1*^ and the heteroallelic combination *mle1/mleγ203*. The slight difference in phenotype distribution observed in the *mle*^*1*^*/mle*^*1*^ flies versus the one reported in Fig 6 is probably due to the difference in temperature at which the flies had been raised (room temperature here versus 25°C in Fig 9).
(PDF)

S1 Table Hairpin length of RNAi stocks. (XLSX)
