# Supplementary figures and images for: The Drosophila Helicase MLE Targets Hairpin Structures in Genomic Transcripts
Source: PLoS Genet. 2016 Jan 11;12(1):e1005761. doi: 10.1371/journal.pgen.1005761 (PMC4710571; doi:10.1371/journal.pgen.1005761)

♂ Hrb87F RNAi

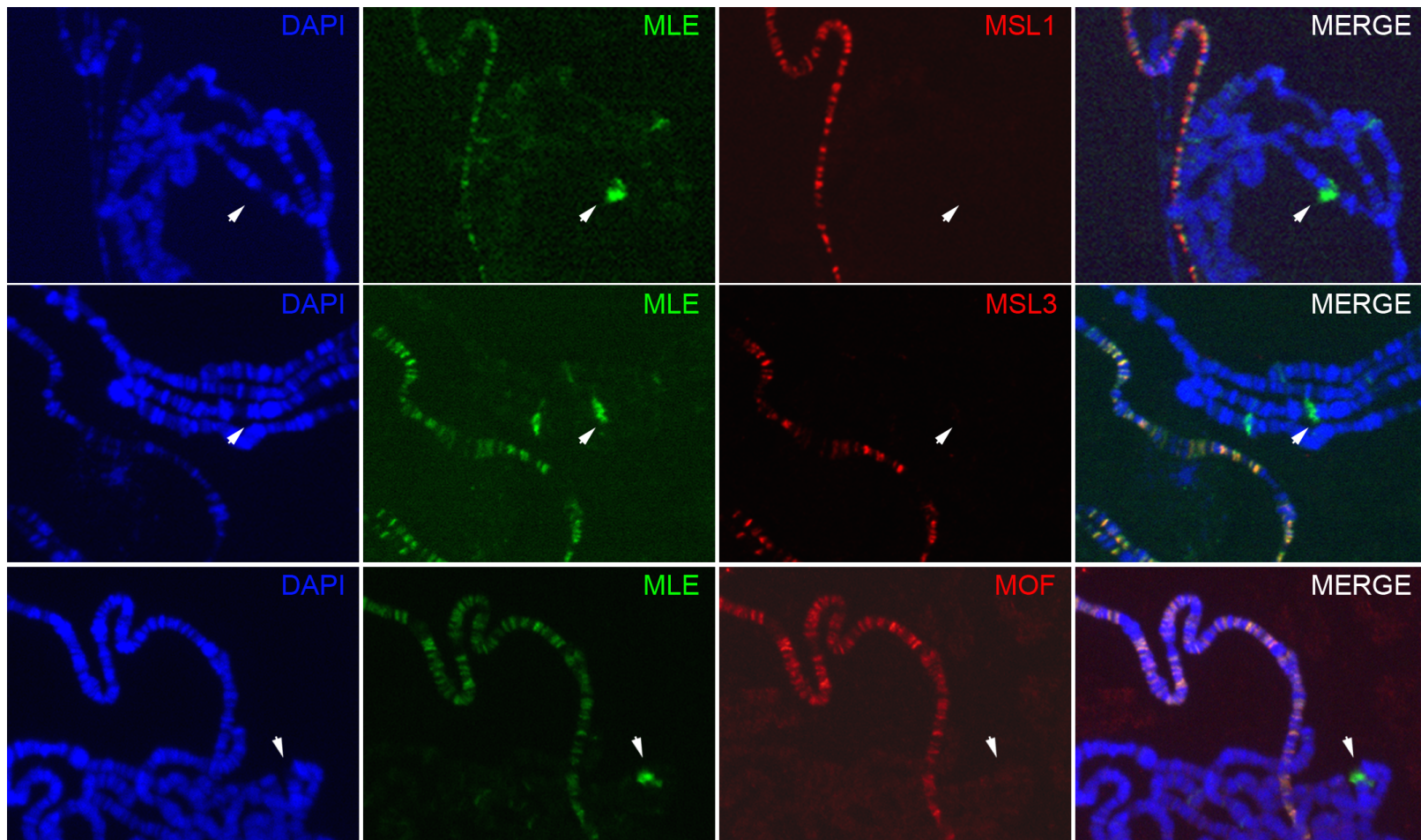

Supplement: S1 Fig — Polytene chromosomes from male larvae expressing a dsRNA targeting Hrb87F (Hrb87F RNAi). Co-staining with Guinea-pig anti-MLE and respectively rabbit anti-MSL1, MSL3 or MOF. The white arrows indicate the integration site of the plasmid. (PDF) [file pgen.1005761.s001.pdf]

♀ Hrb87F RNAi

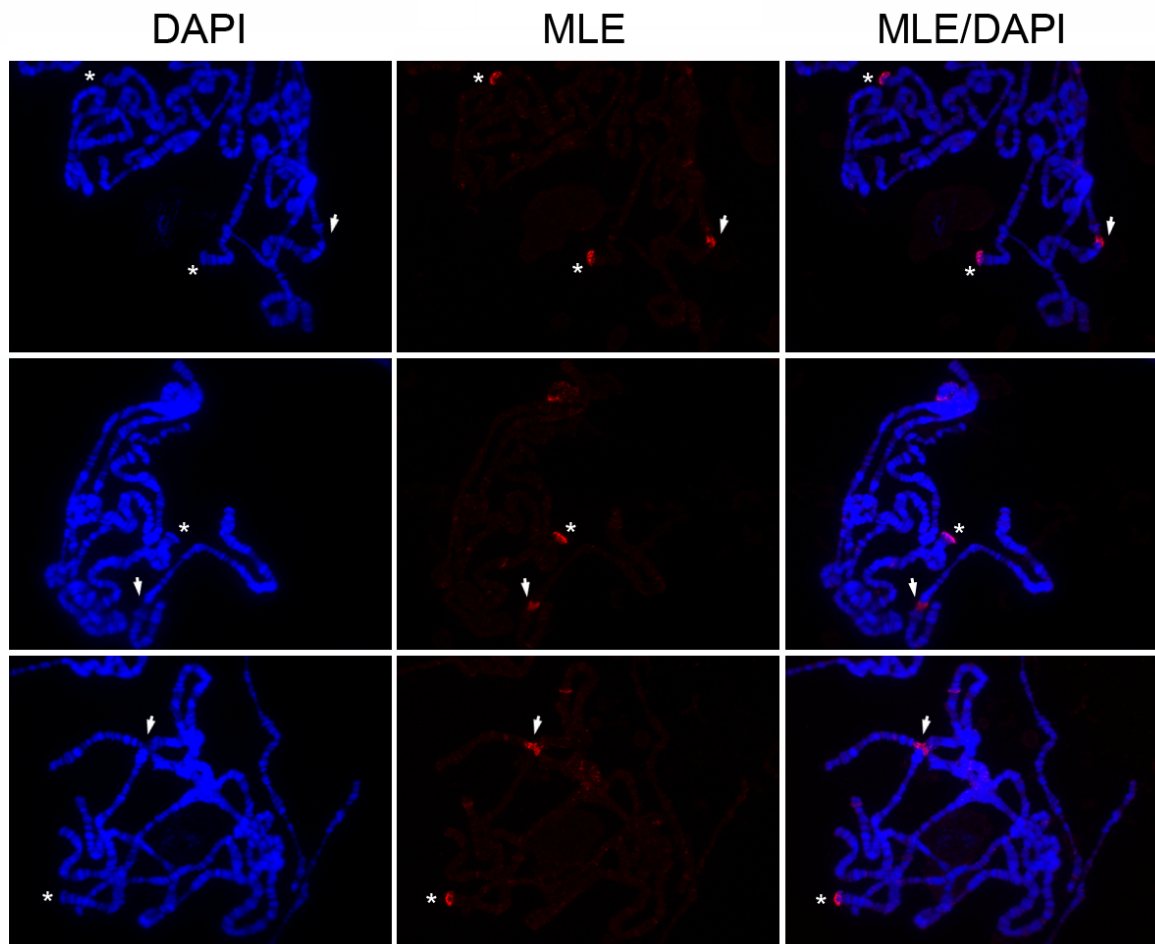

Supplement: S2 Fig — Polytene chromosomes from female larvae expressing a dsRNA targeting Hrb87F (Hrb87F RNAi). MLE staining results in a clear signal at the integration site of the plasmid, indicated by a white arrow, and at the 3R telomere, indicated by an asterisk. (PDF) [file pgen.1005761.s002.pdf]

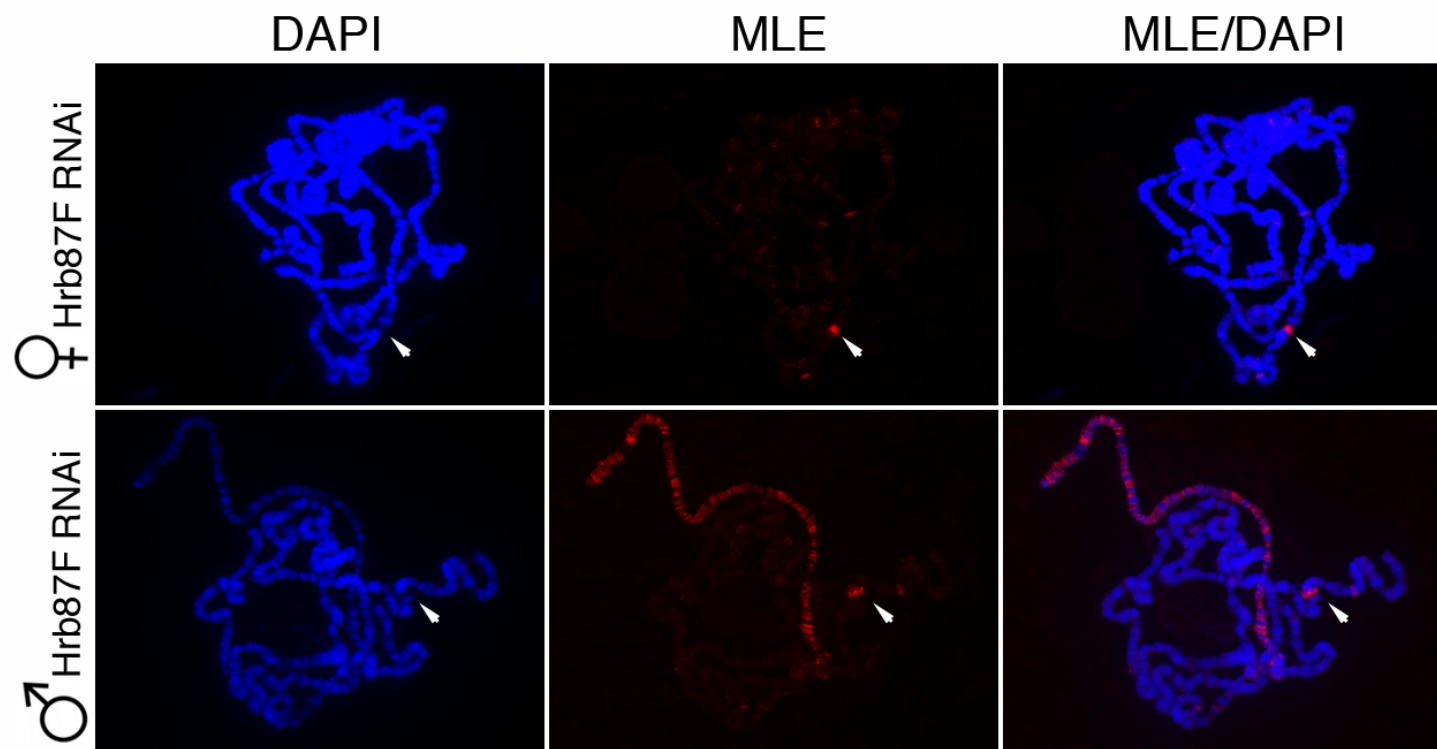

Supplement: S3 Fig — Polytene chromosomes from female and male larvae in which the expression of a dsRNA targeting Hrb87F (Hrb87F RNAi) has been induced by an Act5C-GAL4 driver on the third chromosome. MLE staining results in a clear signal at the integration site of the plasmid expressing the dsRNA. The white arrows indicate the plasmid integration site. (PDF) [file pgen.1005761.s003.pdf]

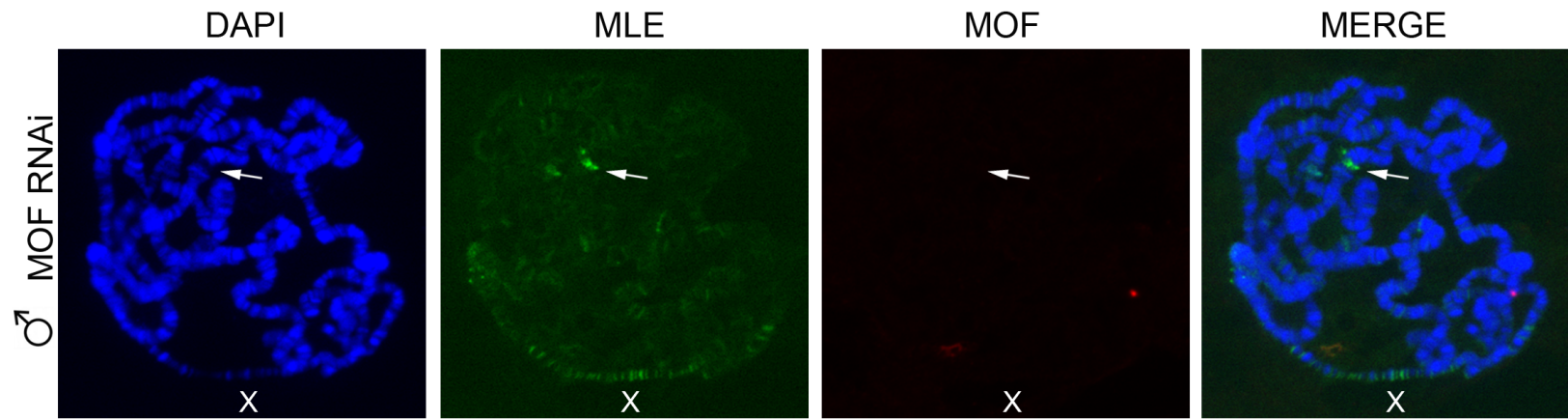

Supplement: S4 Fig — Polytene chromosomes from male RNAi larvae co-stained with anti-MLE and anti-MOF. MLE is present at the integration site of the plasmid (white arrow) and at the X-chromosome in both Hrb87F RNAi and MOF RNAi larvae. MOF is present on the X-chromosome of Hrb87F RNAi larvae while it is completely absent in MOF RNAi larvae, indicating that it was successfully knocked down. (PDF) [file pgen.1005761.s004.pdf]

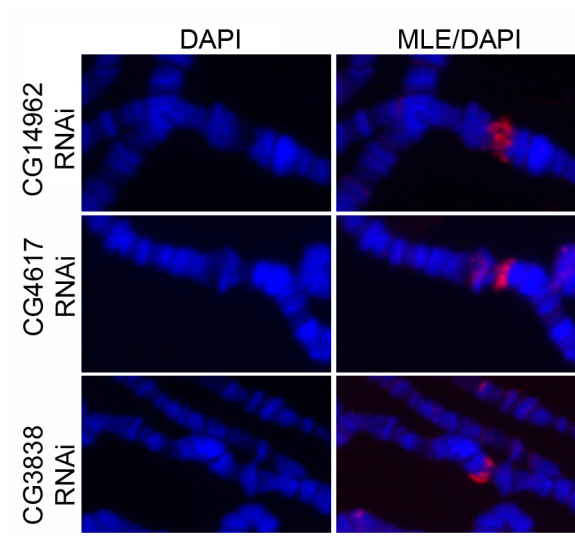

Supplement: S5 Fig — MLE staining of polytene chromosomes from female larvae expressing three different dsRNA inserted in a pValium10 plasmid. (PDF) [file pgen.1005761.s005.pdf]

**A**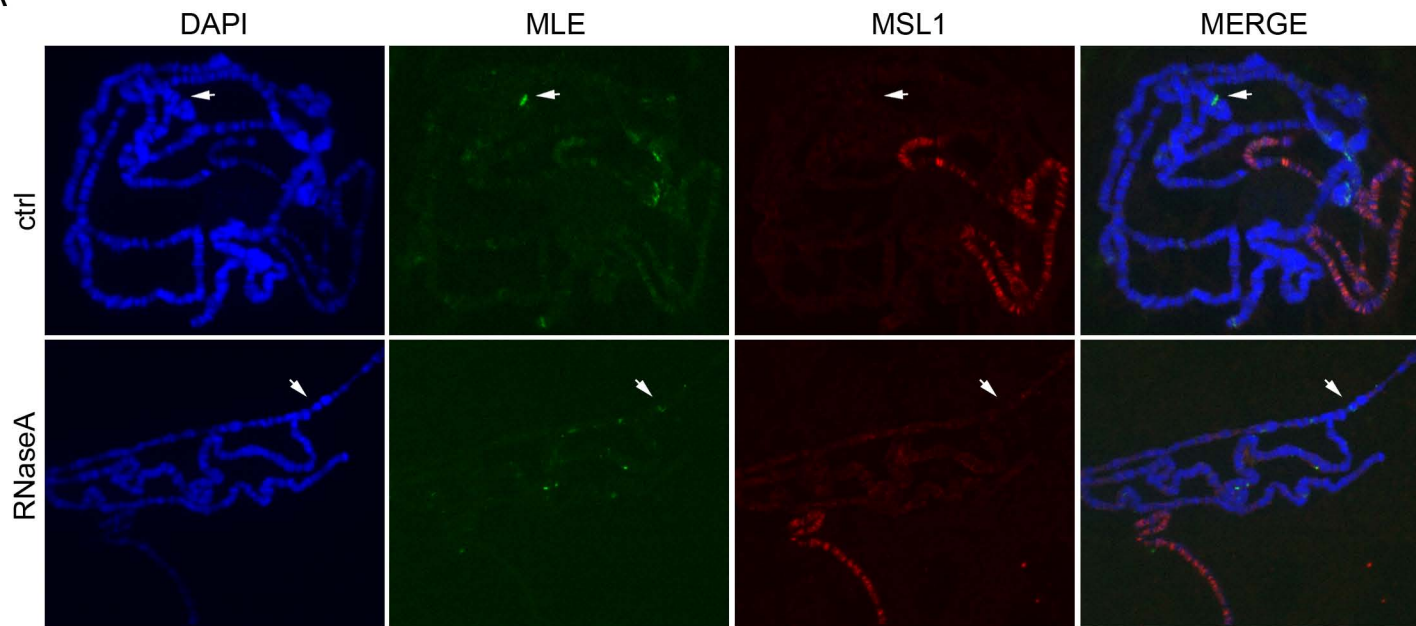**B**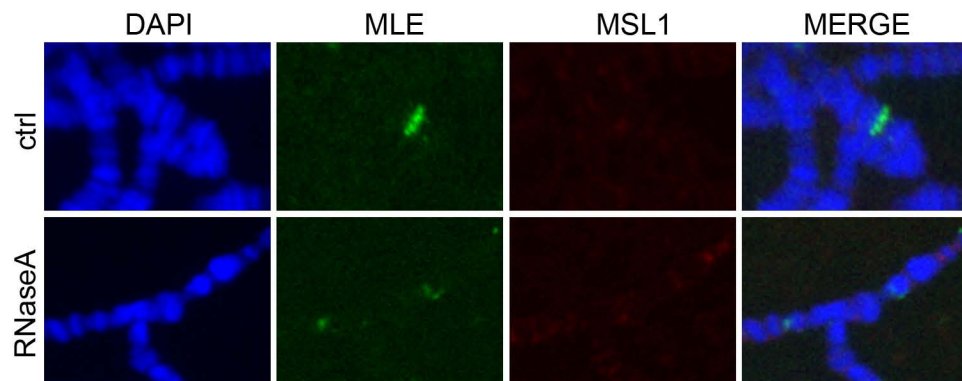**C**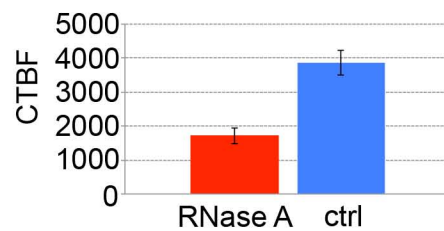

Supplement: S6 Fig — (A) MLE and MSL1 co-staining of polytenes chromosome from male larvae expressing a dsRNA targeting Hrb87F induced by an Act5C-GAL4 driver. Incubation with RNaseA reduces MLE’s enrichment at the site of hairpin transcription. The white arrows indicate the integration site of the plasmid. (B) Magnification of the area indicated by the arrow in portion (A) of the figure. (C) Quantitative analysis of fluorescence levels. MLE signal at the integration site of the plasmid, expressed in terms of corrected total band fluorescence (CTBF), is significantly reduced after RNase A treatment (p value <0.001). The analysis was performed on 6 polytene chromosomes treated with RNase A and 8 control chromosomes. (PDF) [file pgen.1005761.s006.pdf]

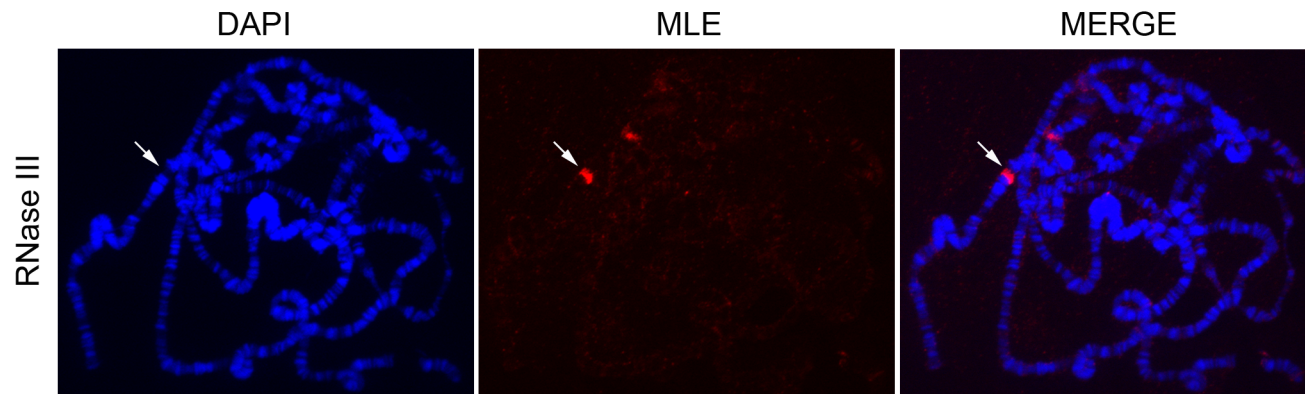

Supplement: S7 Fig — MLE staining of polytenes chromosome from male larvae expressing a dsRNA targeting Hrb87F induced by an Act5C-GAL4 driver. Incubation with RNase III does not appear to affect MLE’s enrichment at site of hairpin transcription. The white arrows indicate the integration site of the plasmid. (PDF) [file pgen.1005761.s007.pdf]

A

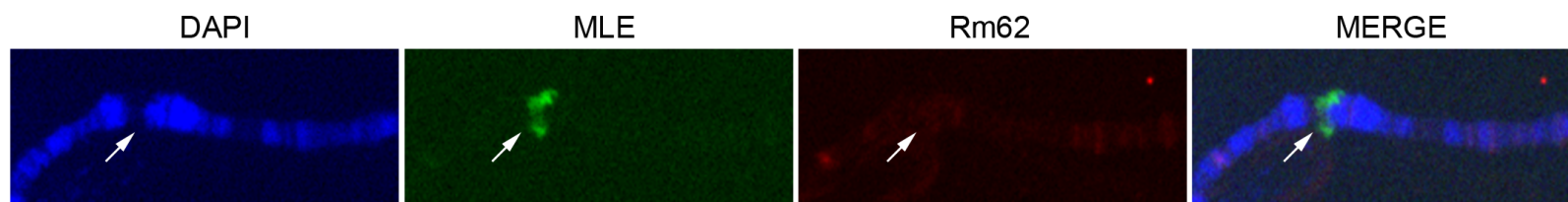

B

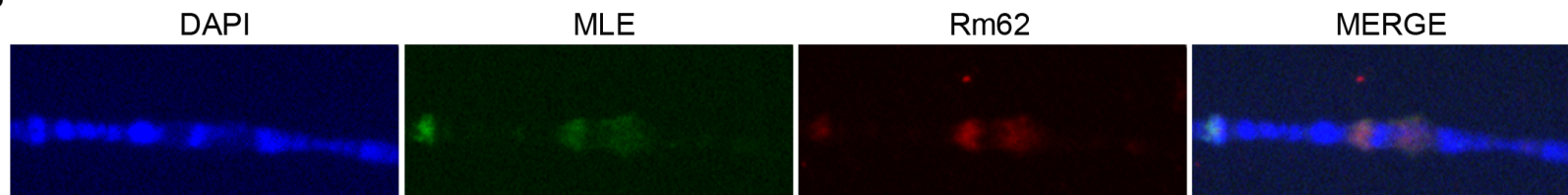

Supplement: S8 Fig — MLE and Rm62 co-staining of polytene chromosomes from female larvae expressing a dsRNA targeting Hrb87F induced by an Act5C-GAL4 driver. (A) MLE is highly enriched at the integration site of the plasmid (indicated by the white arrow) while Rm62 does not appear to be enriched at the same site. (B) MLE and Rm62 are both enriched at developmental puffs. (PDF) [file pgen.1005761.s008.pdf]

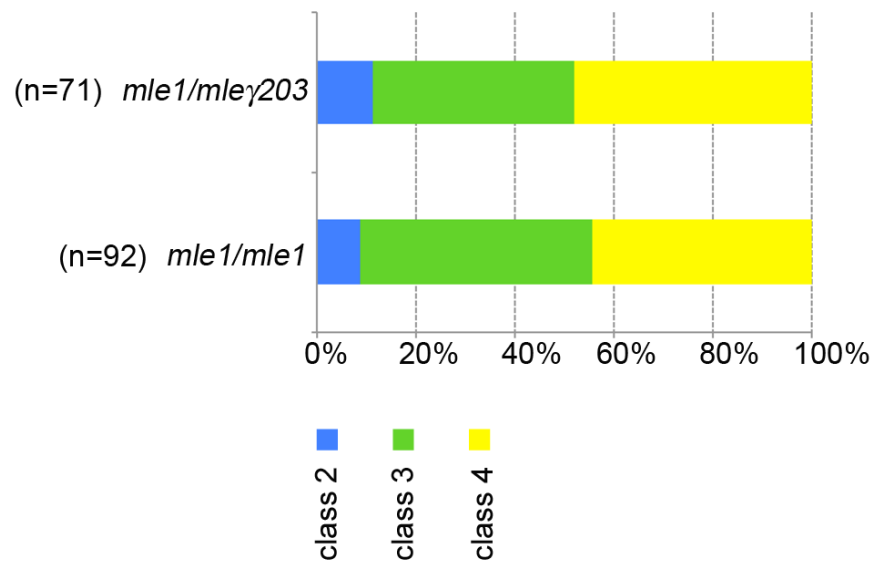

Supplement: S9 Fig — Wing phenotypes from female flies, raised at room temperature, in which Notch dsRNA expression is induced by C96-GAL4. No difference is observed between the homozygous mutant mle1/mle1 and the heteroallelic combination mle1/mleγ203. The slight difference in phenotype distribution observed in the mle1/mle1 flies versus the one reported in Fig 6 is probably due to the difference in temperature at which the flies had been raised (room temperature here versus 25°C in Fig 9). (PDF) [file pgen.1005761.s009.pdf]
